# Dual effect of *Plasmodium*-infected erythrocytes on dendritic cell maturation

**DOI:** 10.1186/1475-2875-9-64

**Published:** 2010-03-01

**Authors:** Esther Bettiol, Daniel Carapau, Cristina Galan-Rodriguez, Carlos Ocaña-Morgner, Ana Rodriguez

**Affiliations:** 1 Department of Medical Parasitology, New York University School of Medicine, 341 East 25th street, New York, NY 10010, USA; 2 Institute of Physiological Chemistry, Medical School, MTZ, Dresden University of Technology, Fiedlerstr. 42, 01307 Dresden, Germany

## Abstract

**Background:**

Infection with *Plasmodium *is the cause of malaria, a disease characterized by a high inflammatory response in the blood. Dendritic cells (DC) participate in both adaptive and innate immune responses, influencing the generation of inflammatory responses. DC can be activated through different receptors, which recognize specific molecules in microbes and induce the maturation of DC.

**Methods:**

Using *Plasmodium yoelii*, a rodent malaria model, the effect of *Plasmodium*-infected erythrocytes on DC maturation and TLR responses have been analysed.

**Results:**

It was found that intact erythrocytes infected with *P. yoelii *do not induce maturation of DC unless they are lysed, suggesting that accessibility of parasite inflammatory molecules to their receptors is a key issue in the activation of DC by *P. yoelii*. This activation is independent of MyD88. It was also observed that pre-incubation of DC with intact *P. yoelii*-infected erythrocytes inhibits the maturation response of DC to other TLR stimuli. The inhibition of maturation of DC is reversible, parasite-specific and increases with the stage of parasite development, with complete inhibition induced by schizonts (mature infected erythrocytes). *Plasmodium yoelii*-infected erythrocytes induce a broad inhibitory effect rendering DC non-responsive to ligands for TLR2, TLR3, TLR4, TLR5, TLR7 and TLR9.

**Conclusions:**

Despite the presence of inflammatory molecules within *Plasmodium*-infected erythrocytes, which are probably responsible for DC maturation induced by lysates, intact *Plasmodium*-infected erythrocytes induce a general inhibition of TLR responsiveness in DC. The observed effect on DC could play an important role in the pathology and suboptimal immune response observed during the disease. These results help to explain why immune functions are altered during malaria, and provide a system for the identification of a parasite-derived broad inhibitor of TLR-mediated signaling pathways.

## Background

Malaria is one of the most lethal infectious diseases worldwide. Acquired immunity to malaria is directed to the blood stage of the parasite and is slow to develop, taking more than five years to develop in individuals living in endemic areas [1]. A better understanding of innate and acquired immunity to *Plasmodium *parasites is required for the development of the development of effective anti-malaria strategies.

Dendritic cells (DC) play a major role in host responses to pathogens, influencing both innate and adaptive immunity, as it is evident by their unique capacity to activate naïve T cells and polarize CD4^+ ^T cell responses. Activation of Toll-like receptors (TLR) induces the maturation of DC, which is required for the generation of effective innate and T cell-mediated responses [2]. DC maturation is associated with the expression at the cell surface of MHC-peptide complexes for antigen presentation to T cells (signal 1), co-stimulatory molecules such as CD40, CD80/B7.1 and CD86/B7.2 (signal 2), as well as secretion of cytokines such as IL-12, that induce T cell polarization [3]. DC maturation can also be induced by activation of the inflammasome [4], which can be triggered by different molecules, such as alum or uric acid [5].

Whether DC mature normally during malaria has been a mater of debate [6]. Studies using DC, either differentiated *in vitro *or directly isolated from infected mice, have shown an inhibition of DC maturation by blood stages of human-infective (*Plasmodium falciparum*) and rodent-infective *Plasmodium *species [7-11]; however, other studies found effective DC maturation, again in both rodent and human models [12-14]. Analysis of malaria infections in mice also produced controversial results showing either activation [15,16] or inhibition of DC function [17,18]. Finally, analysis of DC during human malaria infections has shown that the frequency of BDCA3+ myeloid DCs was significantly increased during acute disease [19], suggesting that the distribution of DC subpopulations is affected during human malaria. Recent studies in mice have contributed to the clarification of these apparent contradictions by demonstrating that the effect of *Plasmodium *on DC is dependent on diverse factors, including the parasite strain [20], the subpopulation of DC studied, the time after infection, and the size of the inoculum [21].

In recent years, extensive characterization of TLR receptors (TLR1 to 12) has been performed, providing new insights on the specificity of innate immunity [22]. Among eukaryotic parasites, diverse TLR ligands have been identified, such as glycophosphatidyl-inositol (GPI) from *Trypanosoma cruzi *[23], lipophosphoglycan from *Leishmania *[24] or profilin from *Toxoplasma *[25]. Two different *Plasmodium*-derived molecules have been identified as activators of antigen-presenting cells through TLR-dependent mechanisms. *Plasmodium*-derived GPI [26] and DNA-covered haemozoin [27,28] activate TLR2 and TLR9, respectively. While GPI induces secretion of TNF by macrophages [26], haemozoin induces maturation of DC and secretion of TNF, IL-12 and IL-6 [27]. In these studies, activation of TLR has been performed using molecules purified from parasites, or else synthetic analogues, but TLR stimulation using whole parasites or lysates was not evaluated.

In this work, the role of *Plasmodium yoelii*-infected erythrocytes in the maturation of DCs was characterized. It was also addressed whether the effects of phagocytosing high amounts of erythrocytes per se was associated with modulation of DC maturation. By assessing the level of expression of co-stimulatory molecules, it is shown that whole *P. yoelii*-infected erythrocytes do not induce a strong maturation response in bone marrow-derived DC *in vitro*. This lack of response was not dependent on parasite stage, viability or time of incubation. Conversely, lysates of infected erythrocytes induced a MyD88-independent activatory response in DC. The inhibition of DC maturation by infected erythrocytes was parasite-specific, since increasing phagocytosis of uninfected erythrocytes by DC did not have the same effect. It was also observed that pre-incubation of DC with infected erythrocytes inhibits the DC maturation response triggered by different TLR ligands, as measured both by the expression of co-stimulatory molecules and the secretion of bioactive IL-12.

## Methods

### Animals and parasites

Balb/C, Swiss Webster and C57BL/6 mice were purchased from Taconic and housed in NYU animal facility according to protocols approved by IACUC. MyD88-deficient C57BL/6 mice were kindly provided by Dr. Elizabeth Nardin (New York University). *Plasmodium yoelii *17XNL parasites were obtained from Swiss Webster mice infected intraperitoneally.

### Culture of mouse bone marrow-derived myeloid DC

**Figure 1:**
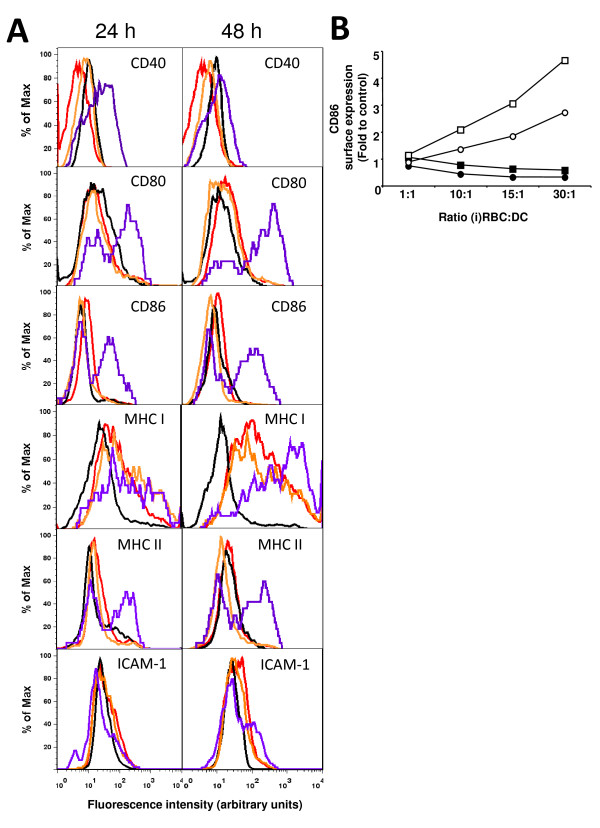
***P. yoelii *lysates, but not whole *P. yoelii*-infected erythrocytes, induce DC maturation *in vitro***. (A) Bone marrow-derived DC were incubated with whole RBC (red), whole P. yoelii-iRBC (black), lysates of RBC (orange), or lysates of iRBC (purple).Ratio of red blood cell (RBC):DC was 30:1, an amount of lysate equivalent to a 30:1 ratio of whole parasites/DC was added to DC. Surface expression of maturation-associated receptors and MHC was determined by FACS after 24 h or 48 h. (B) DC derived from wt (circles) or MyD88-deficient (squares) mice were incubated with lysates of RBC (black symbols) or *P. yoelii*-iRBC (white symbols) at the indicated ratios of RBC:DC. The ratio indicates the amount of RBC or iRBC from which the lysate was derived. Expression of CD86 was determined by FACs after 24 h.

DC were generated from bone marrow precursors as previously described [29]. Briefly, bone marrow cells from the femur and tibia of BALB/c mice of 8-10 weeks of age were harvested and cultured for 8 to 10 days in 80% complete DMEM (containing 10% FBS, penicillin 100 U/ml, streptomycin 0.1 mg/ml, glutamine 0.292 mg/ml), and 20% of GM-CSF (Granulocyte/Macrophage colony-stimulating factor) conditioned medium (medium from cultures of AG8653 myeloma cells that are transfected with GM-CSF). DC culture reproducibly yielded more than 85% of CD11c+ cells. DC from BALB/c, Swiss-Webster or C57BL/6 mice were used in this study and similar results were observed with DC from both mouse strains. All experiments were performed with DC derived from Swiss Webster mice, except for the results shown in Figure 1A, where the staining of MHC-I and MHC-II required the use of DC from BALB/c mice and Figure 1B, where MyD88-deficient DC can only be obtained from C57BL/6 mice. At least one of the confirmatory experiments for all results obtained with Swiss-Webster DC, was performed using BABL/c-derived DC. These results are not shown and are noted in the figure legends as "Figures are representative of one out of three (or two) independent experiments." No differences were found between the responses of DC derived from the different mice strains.

### Isolation of parasites and erythrocytes components

Schizonts were isolated from the interphase of 45% Percoll gradient spun for 15 minutes at 450 g at RT. Preparations of over 90% schizonts were obtained. Fractions comprised of *Plasmodium *schizont, trophozoites or else mixed uninfected erythrocytes+ ring stages, were isolated using a multilayer Percoll gradient (65%-45%) as modified from Kariuki *et al *[30]. Briefly, *P. yoelii *schizonts were collected from the PBS-45% interphase, while trophozoites were collected from the 45-65% interphase, and uninfected erythrocytes+rings from the pellet.

*Plasmodium yoelii *and control lysates were prepared by freeze-thawing five times schizonts or uninfected erythrocytes, respectively. An amount of lysate equivalent to a 30:1 ratio of whole parasites/DC was added to DC. Food vacuoles were isolated following the protocol described by Saliba *et al *[31]. Schizonts were saponized for a few seconds by resuspending them in a solution of 0.5 mg/ml saponin (Sigma-Aldrich), followed by two washes with PBS. Food vacuoles isolated from 0.3 × 10^9 ^to 1 × 10^9 ^saponized schizonts were added per 10^6 ^DC. To fix parasites, schizonts were rinsed in PBS, resuspended in 4% paraformaldehyde and incubated for 15 minutes at room temperature. Finally, schizonts were rinsed three times with PBS. To induce phosphatidyl-serine flipping, control erythrocytes were treated with 1 μM ionomycin in DMSO (Sigma-Aldrich) for 30 minutes at 37C, rinsed and plated on cells. To isolate haemoglobin, blood of uninfected Swiss Webster mice was centrifuged at 100 g twice to remove platelets. The blood was then passed through a Plasmodipur filter (Euro-Diagnostica, Arnhem, The Netherlands) to remove leucocytes. Erythrocytes were then lysed in water for 5 min on ice. PBS 10× was added to a final 1× concentration and the erythrocytes ghosts were removed by centrifugation at 16,000 × g for 20 minutes twice. Protein concentration was measured using Bradford dye reagent (Bio-Rad) according to the manufacturer's instructions.

### Co-cultures of bone marrow-derived DC and erythrocytes

After differentiation, DC were plated at 10^6^/ml in complete DMEM supplemented with 10% GM-CSF conditioned medium. DC were stimulated with either *P. yoellii *17XNL-infected or uninfected erythrocytes (isolated from Swiss-Webster mice) at a 1:30 DC to erythrocytes ratio, unless indicated otherwise. For experiments where parasites were incubated for definite amounts of time, erythrocytes were removed by rinsing cells five times with PBS, while the adherent DC remain in culture.

### TLR ligands

DC were stimulated with the following TLR ligands: LPS from *Salmonella typhimurium *(Sigma-Aldrich) for TLR4, CpG oligodeoxynucleotides (ODN 2395) for TLR9, Zymosan from *Saccharomyces cerevisiae *(Sigma-Aldrich) for TLR2, Polyinosinic-polycytidylic (PolyI:C) for TLR3, Flagellin from *Salmonella typhimurium *for TLR5, Pam3CysSerLys4 (P3C) for TLR1/TLR2 and loxoribine for TLR7 (all from Invivogen). Concentrations used are indicated in figure legends.

### Analysis of expression of co-stimulatory molecules by flow cytometry

After harvesting, DC were incubated for 30 min on ice with Fc block (BD Biosciences, clone 2.4G2) at 1:500 dilution in PBS+1% Bovine Serum Albumin. DC were then stained for 1 h on ice in PBS+1% Bovine Serum Albumin with the following fluorescently labeled antibodies at 1:100 dilution: CD11c (Biolegend, clone N418 or BD Biosciences, clone HL3), CD40 (BD Biosciences, clone 3/23), and CD86 (BD Biosciences, clone GL1). Fluorescently labeled isotype controls were used in control samples and analysed in parallel to exclude nonspecific binding. To assess cell viability, 7-amino-actinomycin D (7-AAD) (BD Biosciences) was added to labeled DC 10 minutes before flow cytometry analysis. For analysis, cells were gated on expression of CD11c and absence of 7-AAD staining. Surface marker expression was assessed for CD86 as the percentage of CD86^high ^cells and as mean fluorescence intensity for CD40. Annexin-FITC (Invitrogen, Molecular Probes) was used according to the manufacturer's instructions. Briefly, 10^6 ^erythrocytes/ml were labeled for 15 min with Annexin-FITC at 1:20 dilution and analysed by flow cytometry shortly after. All flow cytometry experiments were performed on a Becton-Dinckinson FACS Calibur and data analysed with Cell Quest or FlowJo software.

### Fluorescent labeling of erythrocytes

Schizonts or erythrocytes were labeled using a protocol modified from Ing *et al *[16]. Briefly, uninfected or else *P. yoelii*-infected erythrocytes diluted in PBS at 10^8 ^erythrocytes/ml were incubated for 15 minutes at 37C with 1 μM CellTrace™ Far Red DDAO-SE (Molecular Probes), rinsed with PBS, incubated further for 45 minutes at 37C in complete medium and rinsed once more with PBS. For phagocytosis assays, labeled erythrocytes were centrifuged for 1 min at 1,000 rpm on DC at a 1:30 ratio and incubated at 37C for 4 h. Cells and erythrocytes were scraped on ice and directly analysed by flow cytometry. DC were gated using the size and morphology parameters (FSC, SSC) and DDAO fluorescence was analysed within the DC population.

### IL-12 production by DCs

Bone marrow-derived DCs were plated as mentioned before and uninfected or *P. yoelii*-infected erythrocytes were added at different ratios of erythrocytes:DC. At different times after the start of erythrocytes incubation, LPS was added at 1 μg/ml for an additional 24 h. At the end of LPS incubation, supernatants from DC/erythrocytes co-cultures were removed and spun at 13,000 g for 10 minutes. Supernatants were used for ELISA for quantification of IL-12p70 (BD Biosciences) according to manufacturer's instructions.

## Results

### *Plasmodium yoelii *lysates, but not whole *P. yoelii*-infected erythrocytes, induce DC maturation *in vitro*

During malaria infection, DC and other immune cells are frequently exposed to parasite materials derived from lysed infected erythrocytes, but also to intact infected erythrocytes, in which case *Plasmodium*-derived TLR ligands will be inside infected erythrocytes. Since immature DC have high phagocytic activity they easily uptake whole *P. yoelii*-infected erythrocytes [9,16]. To assess the effect in DC maturation of lysed and whole intact *P. yoelii*-infected erythrocytes, DC differentiated *in vitro *were incubated with infected erythrocytes at the schizont stage, or else lysates corresponding to the same amount of infected erythrocytes, as well as the respective uninfected controls.

Whole infected erythrocytes did not induce increases in surface expression of any of the maturation markers, while their lysates were very active in triggering a DC response (Figure 1A). To determine whether the maturation induced by *P. yoelii *lysates is mediated through a MyD88 dependent pathway, DC derived from wild type or MyD88-deficient mice were used. It was found that *P. yoelii *lysates induce DC maturation through a MyD88 independent pathway (Figure 1B). There are three characterized inflammatory molecules in *Plasmodium*-infected erythrocytes: glycosylphosphatidylinositol (GPI) anchors [32], a parasite-induced polymer of degraded haem called haemozoin bound to parasite DNA [27,28] and *Plasmodium*-derived uric acid [33,34]. Since MyD88 is required for the activation mediated by GPI and DNA-covered haemozoin (TRL 2 and 9, respectively), this result suggests that the maturation induce by *P. yoelii *lysates may be mediated by uric acid, which is independent of MyD88 [33].

### Whole *P. yoelii*-infected erythrocytes do not induce DC maturation *in vitro*

**Figure 2:**
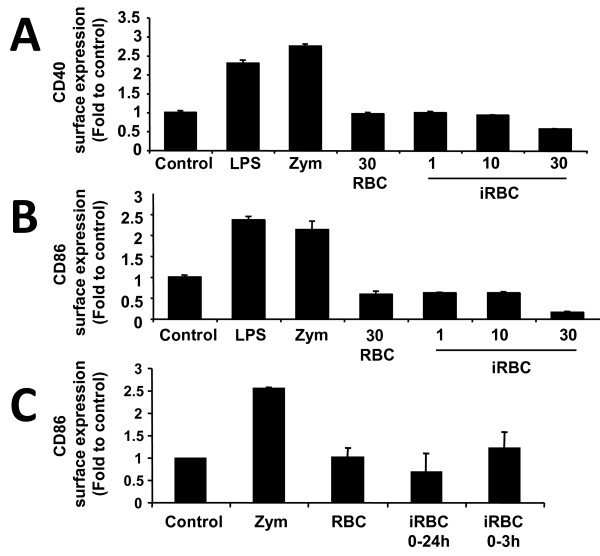
**Whole *Plasmodium*-infected red blood cells (iRBC) do not induce DC maturation *in vitro***. Bone marrow-derived DC were incubated for 24 h in different conditions. Ratio of red blood cell (RBC):DC is 30:1 unless specified otherwise. Expression of CD86 and CD40 analyzed by flow cytometry is expressed as fold-to-control (DC cultured alone). LPS (*Salmonella typhimurium*) and Zymosan (*Saccharomyces cerevisiae*) were used as positive controls for DC maturation. CD40 (A) and CD86 (B) expression after incubation with RBC or *P. yoelii*-iRBC at different ratios. (C) Expression of CD86 at 24 h of culture when DC were co-incubated with iRBC for a short time (3 h pulse) and then washed, or for the whole 24 h. Data correspond to the mean of triplicate samples ± standard deviation. Figures are representative of one out of three independent experiments.

To analyse in more detail the lack of DC maturation observed with whole infected erythrocytes, surface expression of CD40 and CD86 was tested in DC incubated at different infected erythrocytes:DC ratios ranging from 1:1 to 30:1 and used TLR ligands such as LPS (TLR4 ligand) and Zymosan (TLR2 ligand) as positive controls (Figures 2A and 2B). Whole infected erythrocytes did not induce increases in surface expression of CD40 or CD86 in DC at infected erythrocytes:DC ratios ranging from 1:1 to 30:1 (Figures 2A and 2B). The effect of pulsing DC with infected erythrocytes for 3 h, followed by washing and further incubation, was also analysed, showing no maturation of DC at 24 h of culture (Figure 1C).

**Figure 3:**
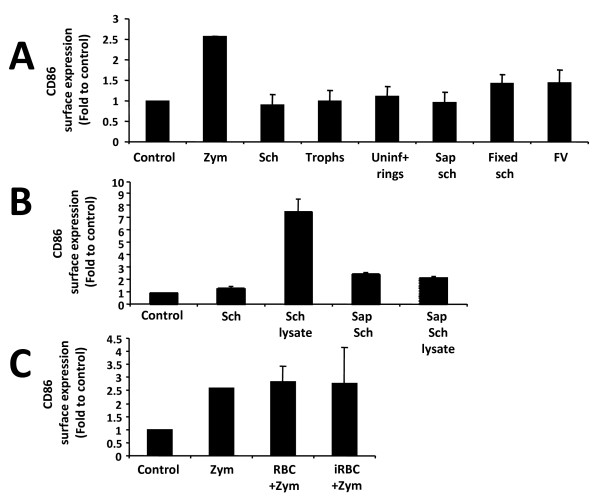
***P. yoelii*-iRBC do not induce DC maturation *in vitro***. Bone marrow-derived DC were incubated for 24 h in different conditions. Ratio of red blood cell (RBC):DC is 30:1 (A) CD86 expression of DC co-incubated with iRBC at the schizont stage (Sch), the trophozoite stage (Trophs), a mix of uninfected RBC and rings (Uninf+rings), fixed iRBC, saponized schizonts or isolated food vacuoles (FV). (B) CD86 expression of DC co-incubated with iRBC at the schizont stage (Sch), its lysate, saponized schizonts (Sap sch) or its lysate. An amount of lysate equivalent to whole parasites was added to DC. (C) CD86 expression of DC co-incubated with RBC or iRBC and zymosan. Data correspond to the mean of triplicate samples ± standard deviation.

As the preparations of *P. yoelii*-purified infected erythrocytes used in this study mainly contain parasites at the schizont stage, it was next investigated if other stages of intra-erythrocytic *P. yoelii *parasites were able to trigger DC maturation. To this aim, schizonts, trophozoites and rings were separated using a multilayer Percoll gradient. Rings were obtained together with uninfected erythrocytes. Each fraction was added to DC at a 30 infected erythrocytes:DC ratio for 24 h. Maturation was not triggered by any of the parasite stages tested (Figure 3A).

To assess the role played by erythrocytes versus parasite-derived components, schizonts were treated with a mild detergent to lyse the erythrocytes membrane allowing for enrichment of isolated parasites and also purified food vacuoles. Isolated parasites did not trigger maturation of DC, neither did food vacuoles (Figure 3A). Lysates of isolated parasites also failed to induce DC maturation (Figure 3B) suggesting that the activatory molecules might be in the cytosol of the infected erythrocytes and not in the parasite itself. However, these molecules could be lost or inactivated in process of purification and lysis of the parasites. Fixed parasites, which should preserve parasite TLR ligands and prevent parasites from releasing newly synthesized metabolites also did not trigger maturation (Figure 3A). It was then tested whether an active parasite-derived inhibitory activity was causing the lack of DC maturation. DC were incubated simultaneously with infected erythrocytes and a strong activator of DC maturation, such as zymosan. It was observed that DC mature in response to zymosan regardless of the presence of infected erythrocytes, when both are added simultaneously (Figure 3C). This suggests that the absence of DC maturation in response to whole *P. yoelii*-infected erythrocytes is a result of lack of activation rather than active inhibition.

### *Plasmodium yoelii*-infected erythrocytes inhibit DC maturation

**Figure 4:**
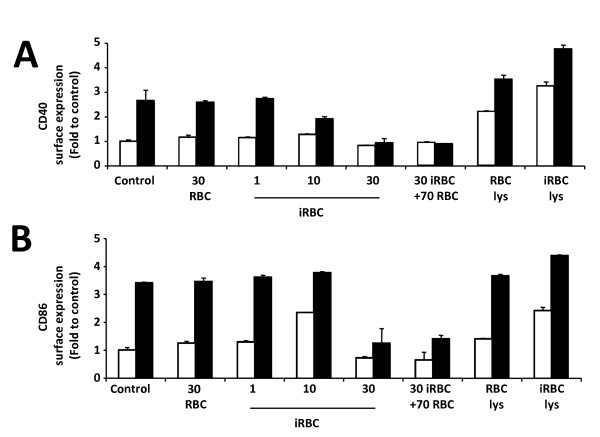
***P. yoelii*-infected erythrocytes inhibit DC maturation in response to the TLR4 ligand LPS**. Bone-marrow derived DC were incubated for 24 h in different conditions and then further stimulated with LPS (black bars) or not (white bars) for 18 h. CD86 and CD40 expression determined by flow cytometry are expressed as fold-to-control (DC alone). CD40 (A) and CD86 (B) expression after incubation of DC for 24 h with RBC or *P. yoelii*-iRBC at different ratios, as well as RBC or iRBC lysates (lys) and with or without LPS for another 18 h. Data correspond to the average of triplicate samples ± standard deviation and figures are representative of one out of two independent experiments.

It was observed that *P. yoelii*-infected erythrocytes do not inhibit DC maturation when added simultaneously with a TLR ligand (Figure 3C). However, pre-incubation of DC with *P. yoelii*-infected erythrocytes inhibits DC maturation induced by LPS [9]. The effect of pre-incubation with whole *P. yoelii*-infected erythrocytes or their lysates on DC maturation was, therefore, compared. DC that had been incubated with uninfected or infected erythrocytes, or else their lysates for 24 h were further incubated with LPS for an additional 18 h. The effect on expression of CD40 (Figure 4A) and CD86 (Figure 4B) was assessed by flow cytometry. This confirmed that whole *P. yoelii*-infected erythrocytes inhibit DC maturation in a dose-dependent manner, with complete inhibition at 30 infected erythrocytes:DC ratio. On the contrary, lysates at similar parasite densities did not inhibit LPS-induced maturation. Interestingly, it was found that with a 10:1 infected erythrocyte:DC ratio partial activation of DC (increased CD86 expression) occurred after 48 h of incubation (Figure 4B), while there was also a decrease in CD40 expression (Figure 4A). This observation, however, was not reproducible, and was found only in two out of five independent experiments. In the other three experiments, no changes in the levels of CD40 and CD86 expression were, as had been previously described [35]. These observations suggest that on certain occasions higher densities of infected erythrocytes (30:1) are required for complete inhibition, and that whole *P. yoelii*-infected erythrocytes can trigger a partial DC maturation phenotype, which is slow to develop (only seen at 48 h, Figure 4B). This phenomenon might be due to lysis of infected erythrocytes after prolonged *in vitro *incubation and would mimic the results obtained when DC are incubated with lysates of infected erythrocytes (Figure 1A).

It was also investigated whether the presence of additional uninfected erythrocytes in the cultures of DC with infected erythrocytes, which resembles more closely the situation *in vivo*, would have an effect on parasite-mediated inhibition of DC maturation. When 70 uninfected erythrocytes:DC were added together with 30 infected erythrocytes:DC, mimicking a 30% parasitaemia infection, a complete inhibition of LPS-induced maturation was observed (Figure 4A, B), suggesting that uninfected erythrocytes do not interfere with the parasite-mediated effect on DC.

**Figure 5:**
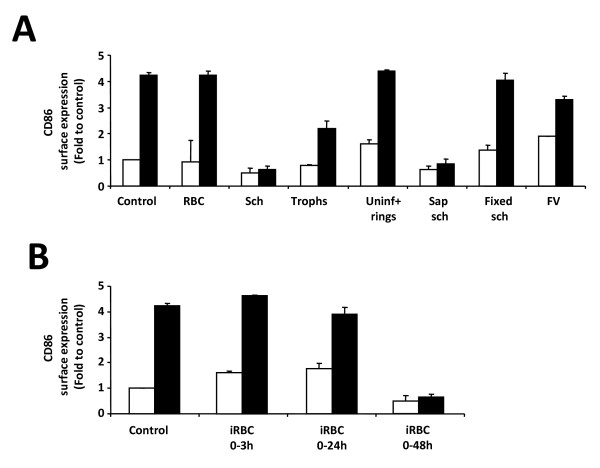
**The schizont stage of *P. yoelii*-infected erythrocytes inhibit DC maturation in response to LPS**. Bone-marrow derived DC were incubated for 24 h in different conditions and then further stimulated with LPS (black bars) or not (white bars) for 18 h. (A) CD86 expression of DC co-incubated with iRBC at the schizont stage (Sch), the trophozoite stage (Trophs), a mix of uninfected RBC and rings (Uninf+rings), fixed iRBC, saponized iRBC or isolated food vacuoles (FV) for 24 h, followed by addition of LPS and incubation for another 18 h. (B) CD86 expression of DC incubated with iRBC for 0-3, 0-24 or 0-48 h, with addition of LPS at 24 h. Data correspond to the average of triplicate samples ± standard deviation and figures are representative of one out of two independent experiments.

It was then assessed whether the intra-erythrocytic stage of parasite development played a role in the inhibition of DC maturation. Each of the parasite stages were added at a similar erythrocyte:DC ratio (30:1). Interestingly, purified *P. yoelii*-infected erythrocytes containing parasites at the trophozoite stage had only a partial inhibitory effect compared to the inhibition induced by the schizont stage. The immature ring-stage parasites had no effect on inhibition of DC maturation (Figure 5A).

When schizonts were extracted from infected erythrocytes before incubation with DC, thereby removing most erythrocyte components, they also induced complete inhibition of LPS-induced maturation (Figure 5A). This observation suggests that there might be an inhibitory factor that is localized in the parasite itself and not in the host erythrocyte. Indeed, a *P. yoelii*-derived soluble factor inhibits the maturation of DC [35].

It was also observed that fixation of the infected erythrocytes in the schizont stage did not inhibit LPS-induced DC maturation (Figure 5A). These observations suggest that metabolically active parasites are required for the observed inhibition of DC maturation to take place.

To assess if the inhibitory effect of *P. yoelii*-infected erythrocytes on DC maturation required continuous presence of infected erythrocytes, the infected erythrocytes were rinsed after 3, or else 24 h of incubation. Unlike the continuous incubation for 48 h, removal of infected erythrocytes allowed maturation of DC to occur in response to LPS (Figure 5B). These results suggest that the inhibitory effect is reversible and that DC can fully mature upon removal of infected erythrocytes. However, the recovery of maturation response in this experiment may be due to removal of molecules produced by metabolically active parasites that are present in the co-culture supernatant and are responsible for the inhibition of DC maturation, rather than removal of the infected erythrocytes themselves.

### Uninfected erythrocytes do not inhibit LPS-induced maturation, even if phagocytosed at a high rate

**Figure 6:**
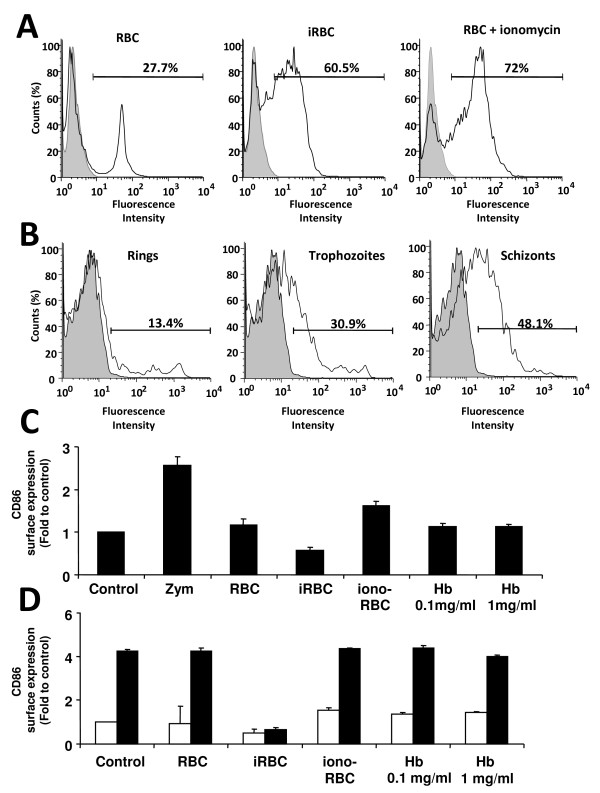
**Red blood cell components did not affect maturation of dendritic cells**. (A, B) To assess the percentage of phagocytosis, uninfected red blood cells (RBC), *P. yoelii*-infected RBC (iRBC) at the schizont stage and RBC treated with ionomycin (A) or different stages of *P. yoelii*-infected RBC (B) were labeled with the fluorescent dye CellTrace™ Far Red DDAO-SE, and incubated for 4 h with DC, before analysis by flow cytometry. The grey curve represents control DC that were not incubated with RBC. (C) CD86 expression analyzed by flow cytometry of DC incubated for 24 h with RBC, iRBC, Ionomycin-treated RBC (iono-RBC) and mouse hemoglobin (Hb). (D) CD86 expression analyzed by flow cytometry of DC incubated for 24 h with RBC, iRBC, Ionomycin-treated RBC (iono-RBC) and mouse hemoglobin (Hb), followed by another 18 h incubation with LPS (black bars) or PBS (white bars). Data correspond to the average of triplicate samples ± standard deviation and figures are representative of one out of three independent experiments.

Macrophage function is decreased by excessive erythrophagocytosis [36], as well as by phagocytosis of *P. falciparum*-infected erythrocytes [37]. In light of previous results, it was possible that the excessive phagocytosis of erythrocytes components could be the cause of the absence of DC maturation and resistance to LPS-induced maturation. As *Plasmodium*-infected erythrocytes are phagocytosed with significantly higher efficiency compared to uninfected erythrocytes [16], control erythrocytes were treated with ionomycin to increase their phagocytosis, since this treatment triggers phosphatidyl-serine flipping and an apoptosis-like phenotype in erythrocytes [38,39]. Using fluorescently-labeled erythrocytes in these experimental conditions, it was possible to confirm that uninfected control erythrocytes were phagocytosed with low efficiency when compared to infected erythrocytes (27.7% and 60.5% respectively, Figure 6A, left and middle panels). After treatment of erythrocytes with ionomycin for 30 min, significant flipping of phosphatidyl-serine to the outer membrane was observed by staining with Annexin-V. As expected, erythrocytes treated with ionomycin were phagocytosed very efficiently, to a level comparable to that of *P. yoelii*-infected erythrocytes at the schizont stage (Figure 6A, right panel).

Interestingly, phagocytosis of infected erythrocytes increases with the maturation stage of the parasite, resulting in very efficient phagocytosis of schizonts, intermediate phagocytosis of throphozoites and reduced phagocytosis of ring stages (Figure 6B). This would be compatible with the progressive modifications that are observed in the membrane of infected erythrocytes during an infection cycle [40]. Since there is a good correlation between the level of DC maturation inhibition (Figure 5A) and the level of phagocytosis (Figure 6B) with the different developmental stages, it was determined whether high levels of phagocytosis of uninfected erythrocytes would affect DC maturation. Ionomycin-treated erythrocytes did not induce DC maturation (Figure 6C) but failed to prevent LPS-induced maturation (Figure 6D), as observed previously with *P. yoelii*-infected erythrocytes. To confirm that the most relevant component of erythrocytes does not interfere with DC maturation, DC were preincubated with haemoglobin isolated from control erythrocytes. DC maturation was not affected, even when used at a concentration as high as 1 mg/ml (Figure 6D). These results exclude the possibility of unspecific effects due to different levels of phagocytosis of infected and uninfected erythrocytes and also confirm the ability of DC to mature even after high levels of erythrophagocytosis.

### Kinetics of the inhibition of DC maturation by *P. yoelii*-infected erythrocytes

**Figure 7:**
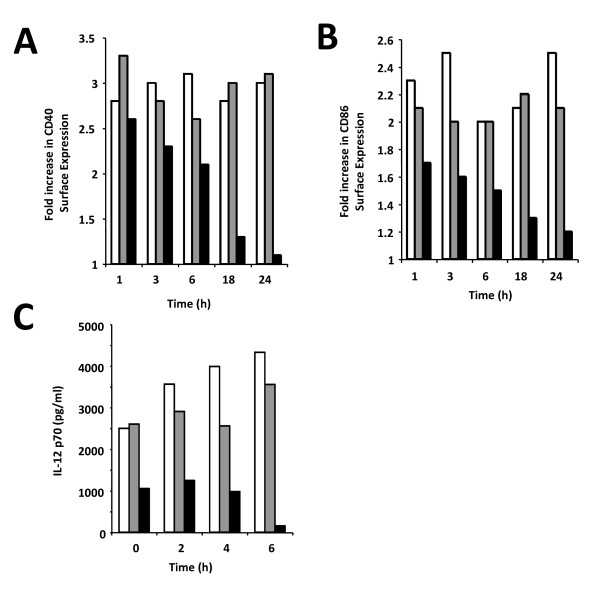
**Inhibition of LPS-induced maturation by *P. yoelii*-infected red blood cells (iRBC) is established between 6 and 18 h**. To investigate the kinetics of the iRBC-induced inhibition of DC maturation, DC were pre-incubated for different times (1 to 24 h) with no RBC (white bars), uninfected RBC at 30:1 (grey bars) or iRBC at 30:1 (black bars), and then pulsed with LPS for 18 h. Cells were then analyzed by flow cytometry for expression of CD40 (A) and CD86 (B). To characterize the kinetics of the iRBC-induced inhibition of cytokine production, DC were pre-incubated for different times (0 to 24 h) with uninfected RBC at 30:1 (white bars) or iRBC at 10:1 (grey bars) or 30:1 (black bars), and then pulsed with LPS 1 μg/ml. Twenty-four hours after addition of LPS, supernatants of DC cultures were collected and IL-12p70 was quantified by ELISA. Data shows a representative experiment out of two independent experiments.

The results indicate that *P. yoelii*-infected erythrocytes inhibit the maturation of DC in a reversible manner. The kinetics of this inhibition was analysed by testing different times of pre-incubation of DCs with infected erythrocytes before the addition of LPS as a maturation stimulus. The unresponsiveness of DC increased gradually with the time of pre-incubation with infected erythrocytes, with a maximal inhibitory response obtained after 24 h of incubation (Figure 7A, B). The expression of IL-12, a cytokine usually secreted upon DC activation, and which is required for induction of Th1 type responses [2], was also assessed. *Plasmodium yoelii*-infected erythrocytes inhibited IL-12 secretion and that, interestingly, the pre-incubation time required to obtain maximal inhibition of IL-12 secretion was shorter compared to the inhibition of surface expression of co-stimulatory molecules, which might reflect a post-transcriptional, rather than a transcriptional, regulatory mechanism (Figure 7C).

### *Plasmodium yoelii*-infected erythrocytes inhibit maturation induced by stimulation of different TLRs

**Figure 8:**
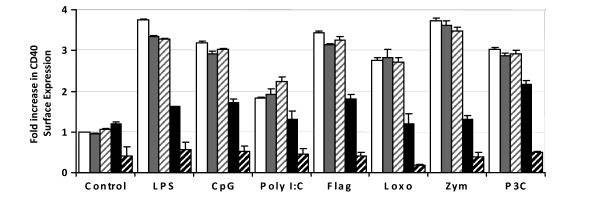
***P. yoelii*-infected red blood cells inhibit maturation of dendritic cells induced by stimulation of different Toll-like Receptors**. To address the effect of *P. yoelii*-infected RBC (iRBC) on DC maturation induced by activation of different TLRs, DC were pre-incubated for 24 h with no RBC(white bars), uninfected RBC(gray bars) or iRBC(black bars) at both 10:1(solid bars) and 30:1(striped bars) ratios, and then pulsed with the following ligands of TLRs for an additional 18 hours: LPS 1 μg/ml, CpG-ODN 1 μg/ml, Zymosan 50 μg/ml, PolyI:C 25 μg/ml, flagellin 0.1 μg/ml and loxoribin 0.5 μM. DC were then analyzed by flow cytometry for expression of CD86. Data correspond to the average of triplicate samples ± standard deviation and figures are representative of one out of three independent experiments.

To investigate if the inhibition of DC maturation induced by *P. yoelii*-infected erythrocytes affected stimulation of DC by different TLRs, DC were pre-incubated with infected erythrocytes for 24 h before stimulation with specific ligands of the following TLRs: TLR1/2 (P3C), TLR2 (zymosan), TLR3 (polyI:C), TLR5 (flagellin), TLR7 (loxoribine), TLR9 (CpG), as well as TLR4 (LPS). For all TLRs studied, the up-regulation of co-stimulatory molecules in DC was severely inhibited by pre-incubation for 24 h with *P. yoelii*-infected erythrocytes in a dose-dependent fashion (Figure 8). These results suggest that the inhibition of DC maturation induced by *P. yoelii*-infected erythrocytes is a broad phenomenon that is not restricted to a specific TLR pathway.

## Discussion

Several pathogens are able to induce maturation of DC *in vitro *in the mouse and human systems. For most of these pathogens, TLR ligands responsible for DC maturation have been isolated, including apicomplexan parasites such as *Toxoplasma *[25], *Trypanosoma *[23], and also *Plasmodium *[26-28]. However, in both *Toxoplasma *and *Plasmodium *cases, several reports suggest that DC maturation can be suppressed by whole parasites either *in vitro *or *in vivo *[6,41].

In this study, it was shown that erythrocytes infected with the rodent malaria parasite *P. yoelii *have two separate effects on DC maturation *in vitro*: (1) parasites induce DC maturation only in certain conditions, which seem to depend on the integrity and dose of infected erythrocytes; also (2) there is a threshold above which whole infected erythrocytes inhibit responsiveness of DC to TLR stimulation after continuous incubation for several hours. This dual response may explain apparently controversial results obtained by different laboratories, since *Plasmodium*-infected erythrocytes from rodent and human species have been found to either activate [12-14], or else inhibit [7-11] DC maturation.

Regarding the activation of DC, it was found that whole *P. yoelii*-infected erythrocytes only induce non-reproducible, weak *in vitro *DC maturation (one out of two markers) and only in limited conditions. On the other hand, lysates of *P. yoelii*-infected erythrocytes induced up-regulation of CD40, CD80, CD86, MHC-I and MHC-II. This last finding agrees with previous studies on TLR ligands purified from *Plasmodium*-infected erythrocytes, such as DNA-covered haemozoin [27,28]. However, the finding that DC maturation induced is independent of MyD88 suggests that another *Plasmodium*-derived activatory molecule, uric acid, may be responsible for the DC maturation observed. The results, therefore, indicate that when the infected erythrocytes lyse before contact with DC, these will undergo maturation. A possible explanation is that *Plasmodium *TLR ligands only become accessible to their receptors on DC after lysis of the erythrocytes when they are released into the medium. Additionally, their activatory effect might be tampered by the presence of other factors in infected erythrocytes that inhibit TLR-induced signaling at higher parasite doses.

It is also described in this study that infected erythrocytes, when present in high densities, prevent DC from up-regulating co-stimulatory molecules and also from secreting IL-12 in response to TLR stimulation. This phenomenon is observed with whole infected erythrocytes, but not with their lysates. A similar dose-dependent *in vitro *effect of *P. falciparum*-infected erythrocytes on DC has been reported [8]. The *Plasmodium*-mediated inhibition of DC maturation is reversible, as responsiveness to TLR ligands is restored if infected erythrocytes are removed, but it is also broad, as expression of co-stimulatory molecules is inhibited in response to all seven TLR ligands tested. Since *P. yoelii*-infected erythrocytes also inhibit the maturation of DC induced by uric acid crystals [35], it appears that *Plasmodium *might be affecting a downstream step in the signaling cascade of DC maturation that is common to different activation pathways.

The fact that the mature stages of *P. yoelii*-infected erythrocytes are required for complete inhibition of DC maturation suggests that the inhibitory factor accumulates along with the asexual cycle inside erythrocytes. It appears that haemozoin does not mediate the observed inhibition, since the effect was not observed with purified food vacuoles.

Complete inhibition of DC maturation requires pre-incubation with *P. yoelii*-infected erythrocytes for at least 18 h. The slow development of a non-responsive state in DC may be related with the time necessary to repress gene expression, but also with a delayed release of the inhibitory factor(s) from infected erythrocytes. Still, this time is significantly shorter than the 48 h needed to observe partial activation of DC (Figure 4B), suggesting that the lysis of infected erythrocytes occurs only during late times of incubation (between 18 and 48 h) and, therefore, the activatory signal is released only after inhibition of DC has been established The fact that DC maturation is not found at high parasite densities might explain why in rodent models of malaria mature DC are found early on at low parasitaemia, but not when the load of infection is higher [42].

High levels of phagocytosis of uninfected [43], as well as infected [16,44], erythrocytes is characteristic of *Plasmodium *blood-stage infections. The study attempted to confirm that the inhibition of DC maturation observed at high ratios of infected erythrocytes is specific of the parasite and not an unspecific effect caused by excessive erythrocyte phagocytosis. Erythrocytes can affect macrophage function [36], including antigen presentation [45], and modify the response to LPS *in vivo *by impairing host defense [46]. High levels of uninfected erythrocyte phagocytosis - induced by ionomycin - did not result in inhibition of DC maturation. Therefore, the uptake of erythrocytes by itself is not sufficient to inhibit DC maturation. Instead, the inhibition of DC maturation is caused specifically by *Plasmodium *parasite, as observed when isolated schizonts are added to DC. The observation that fixed infected erythrocytes are not able to inhibit DC maturation suggests that active parasite metabolism is required for such an effect. Recently, the inhibition of DC maturation by a soluble factor derived from *Plasmodium *infected erythrocytes [35] and by free *Plasmodium *merozoites has been described [47]. Similarly, there was a strong inhibitory activity mediated by isolated schizonts, the parasite stage preceding the release of free merozoites into circulation.

The data presented here indicate that the effects of *Plasmodium *blood stages on DC are very different depending on the integrity of infected erythrocytes. The human parasite *P. falciparum *induces a positive modulation or priming of TLR responses, which in turn results in increased inflammatory cytokine responses after subsequent stimulation. This is not observed when DC are incubated with other microbes, which suppress subsequent TLR responses [48]. It is possible that this unique characteristic of *Plasmodium *is related to the modulation of DC maturation observed here. Even if inflammatory cytokines are produced by DC in response to *P. yoelii*-infected erythrocytes [34], DC do not acquire a mature phenotype. This lack of maturation may account for the possibility of subsequent stimulation of cytokine production that would be suppressed after regular DC maturation.

During a malaria infection, DC probably encounter intact infected erythrocytes, but also lysed erythrocytes after each erythrocytic infection cycle. The two phenomena observed - induction of DC maturation by lysates and inhibition of DC maturation by infected erythrocytes - probably take place during a malaria infection, where high concentrations of infected erythrocytes are reached in organs where parasites accumulate [49,50]. Which outcome prevails might be determined by the amount of infected erythrocytes that accumulate around DC and also by their integrity. These factors will vary with parasitaemia and also cytoadherence properties, leading to changes in the maturation status of DC throughout infection, as suggested before [42]. Indeed, analysis of the whole myeloid DC population from spleens of malaria-infected mice shows a transcriptomic profile very divergent from typically mature DC [17].

Interesting questions are raised by the dual effect of *Plasmodium *in DC maturation and its likely role in the orchestration of the immune response to the parasite. Understanding of the mechanism of inhibition of DC maturation induced by the parasite would help understand not only the regulation of gene expression in DC, but also the effect of modulated DCs on regulating CD4^+ ^and CD8^+ ^T cells.

## Conclusions

*Plasmodium*-infected erythrocytes induce opposite effects on DC: lysed infected erythrocytes induce the maturation of DC, while intact infected erythrocytes induce a broad inhibition of DC maturation by different TLR ligands. These observations explain apparently contradictory findings previously published in this field and also provide an insight into the complexity of the immune response generated by *Plasmodium *in the host.

## List of abbreviations

(DC): Dendritic cells; (TLR): Toll-like Receptors; (GPI): glycophosphatidyl-inositol; (GM-CSF): Granulocyte/Macrophage colony-stimulating factor; (iRBC): Infected Red Blood Cell; (LPS): Lipopolysaccharide.

## Competing interests

The authors declare that they have no competing interests.

## Authors' contributions

EB and DC carried out the DC maturation experiments, conceived the study and helped write the manuscript, CGR carried out the phagocytosis of infected erythrocytes, COM carried out the kinetics of DC inhibition, AR conceived the study and helped write the manuscript. All authors read and approved the final manuscript.
